# Impaired consciousness and decreased glucose concentration of CSF as prognostic factors in immunocompetent patients with cryptococcal meningitis

**DOI:** 10.1186/s12879-020-4794-5

**Published:** 2020-01-22

**Authors:** Chen Zhang, Zheren Tan, Fafa Tian

**Affiliations:** Department of Neurology, Xiangya Hospital, Central South University, 87 Xiangya Road, Changsha, 410008 China

**Keywords:** Cryptococcal meningitis, Clinical features, Prognostic factors, HIV negative, Immunocompetent patients

## Abstract

**Background:**

Cryptococcal meningitis (CM) is the most common fungal infection of the central nervous system and has high morbidity and mortality. Almost studies about prognostic factors have largely focused on the immunocompromised population rather than immunocompetent patients. So that we sought to conduct a retrospective study to determine prognostic factors which predict the outcomes in immunocompetent patients with CM.

**Methods:**

We retrospectively collected and analyzed the demographic and clinical data of 76 apparently immunocompetent patients with cryptococcal meningitis from January 2003 to June 2019 in China. The clinical outcome was graded by the Glasgow outcome scale (GOS) at discharge, and patients were divided into good (score of 5) and unfavorable (score of 1–4) outcome groups, potential prognostic factors were analyzed.

**Results:**

Non-parametric test confirmed that unfavorable outcome was associated with lower glucose level of CSF(*P* = 0.001), and Pearson’s χ2 analysis confirmed that unfavorable outcome was associated with opening pressure of CSF(>300mmH20, *P* = 0.038), impaired consciousness (*P* = 0.001), hydrocephalus(*P* = 0.045), and Shunt surgery (*P* = 0.045), and then multiple logistic regression analysis confirmed that impaired consciousness(*P* = 0.015) and lower glucose concentration of CSF(*P* = 0.012) increased the likelihood of unfavorable outcome in CM patients.

**Conclusion:**

Impaired consciousness and decreased glucose concentration of CSF were independently prognostic factors which predict the unsatisfactory outcome in immunocompetent patients with CM.

## Background

Cryptococcal meningitis (CM) is the most common type of chronic infectious meningitis caused by *Cryptococcus neoformans* [1, 2], the reservoirs of them are mainly pigeon or other bird droppings [3]. The delay in diagnosis and treatment result in a high morbidity and a mortality rate [4–6]. majority of cryptococcosis have occurred in HIV-positive patients, but it is also been found in cases with comorbidities that result in immunosuppression, such as hematological malignancies, solid-organ transplant recipients, and in patients on chronic corticosteroid or other immunosuppressive therapies. However, most patients with CM are immunocompetent in china. Some reports revealed that 10–40% of HIV-negative patients with CM have no apparent immune deficiency [7–9]. But most studies about prognostic factors have largely focused on the immunocompromised population, so that clinical characters and prognostic factors of immunocompetent patients are not well analyzed.

We sought to conduct a retrospective study to characterize clinical features, laboratory findings, imaging findings and determine potential prognostic factors which predict the outcomes in immunocompetent patients with CM.

## Methods

We retrospectively reviewed 116 patients with CM from January 2003 to June 2019 in the Department of Neurology, Xiangya Hospital, Central South University, China.

The diagnostic criteria for cryptococcal meningitis was based on the clinical features and findings of cerebrospinal fluid (CSF) after lumbar puncture. Besides, T-SPOT, tuberculosis ELISA, AFB stain, culture in CSF were performed to rule out the possibility of tuberculosis.

As we all know, the following situations that are correlated with immunodeficiency will be excluded: History of autoimmune disorders, long-term glucocorticoids or other immunosuppressive therapies, patients with idiopathic CD4 T-cell lymphopenia, HIV infection, malignant tumor, hepatic cirrhosis, end-stage renal failure or diabetes. So that patients without these situations will be regarded as immunocompetent.

In this study, we collected demographic data, major symptoms and signs, findings of routine neuroimaging and laboratory findings. The MRI scan, performed on a 1.5 T MRI scanner (Signa GE, Milwaukee, USA), was independently reviewed by radiologists.

At discharge, the clinical outcome was graded by the Glasgow outcome scale (GOS). Score of 1–4, which indicates death, vegetative status, severe and moderate disability, was considered “unfavorable” clinical outcomes. Score of 5, which indicates mild or no disability was considered “good” outcomes. Informed consent was obtained from patients or their guardians.

All statistical analyses were performed using the Statistical Package for IBM SPSS Statistics for Windows (version22.0, Chicago, IL, USA). The demographic and clinical data between the good and poor outcome groups were compared. The quantitative variables were compared using two-sample t-test for parametric data and Mann Whitney U test for non-parametric data. The qualitative variables were compared using Pearson’s χ2 or Fisher exact test, as appropriate. Finally, potentially prognostic factors for predicting the clinical outcome of CM were identified using a multiple logistic regression model. *P*-value < 0.05 was considered statistically significant.

## Results

Overall, a total of 76 apparently immunocompetent patients with CM were included in this study. The median onset age of patients was 50 (range 16, 77) years old. Headache, fever, vomiting were the three most common symptoms. Demographics and clinical features were shown in Table 1.The median white blood cell (WBC) count in the blood was 8.4 (interquartile range 6.6, 11.8) × 10^9^/L. 85.5%(65/76) of the patients had abnormal CSF opening pressure(>180mmH2O), in which 51.3%(39/76) of patients were higher than 300 mm H2O. The median WBC count in the CSF was 50(IQR 18.5, 122.5) 10^6^/L, The median CSF glucose concentration was 1.66(IQR 0.80, 2.85) mmol/L. The median CSF chloride concentration was 117.0 (IQR 113.7, 120.3) mmol/L. The median CSF protein concentration was 0.85 (IQR 0.54, 1.57) g/L. The sensitivity of the CSF India ink test and culture in our study were 86.8 and 8.7%, respectively.34.2% (26/76) of patients shown positive india ink test and culture. Laboratory data are presented in Table 2.

**Table 1:** Demographic and clinical profile of apparently immunocompetent patients with Cryptococcal meningitis

Variable	Value
Gender, M/F	50/26 (66%/34%)
Age at onset (years)	50.0 (40–62)
interval from onset to antifungal treatment (day)	29 (15,40)
duration of antifungal treatment (day)	31 (12.5, 54.5)
Am B administration	69 (90.8%)
Shunt surgery	11 (14.5%)
Main symptoms and signs	
Headache	71 (93.4%)
Fever	48 (63.2%)
Vomiting	38 (50.0%)
Impaired consciousness	15 (19.7%)
Visual disturbance	15 (19.7%)
Seizures	9 (11.8%)
Limb weakness	7 (9.2%)
Altered mentation	7 (9.2%)
Hearing impairment	2 (2.6%)
Meningeal irritation positive	45 (59.2%)

Data are n (%) or median (interquartile range); *Am B* Amphotericin B, *WBC* White blood cell, *CSF* Cerebrospinal fluid

**Table 2:** laboratorial findings and Neuroimaging of apparently immunocompetent patients with Cryptococcal meningitis

Variable	Value
Blood WBC count(10^9^/L)	8.4 (6.6, 11.8)
CSF	
Opening pressure(>180mmH2O)	65 (85.5%)
WBC count(10^6^/L)	50 (18.5, 122.5)
Elevated WBC counts	69 (90.8%)
Glucose (mmol/L)	1.66 (0.80, 2.85)
Increased glucose level	56 (73.7%)
Chloride (mmol/L)	117.0 (113.7, 120.3)
Increased chloride level	55 (72.4%)
Protein (g/L)	0.85 (0.54, 1.57)
Increased protein level	62 (81.6%)
India ink test positive	66 (86.8%)
Culture positive	37 (48.7%)
Neuroimaging	
Dilated Virchow-Robin spaces	50 (65.8%)
Parenchymal lesions	41 (53.9%)
meningeal enhancement	16 (21.1%)
Hydrocephalus	11 (14.5%)
Gelatinous pseudocyst	7 (9.2%)

Data are n (%) or median (interquartile range); *Am B* Amphotericin B, *WBC* White blood cell, *CSF* Cerebrospinal fluid

**Fig. 1:**
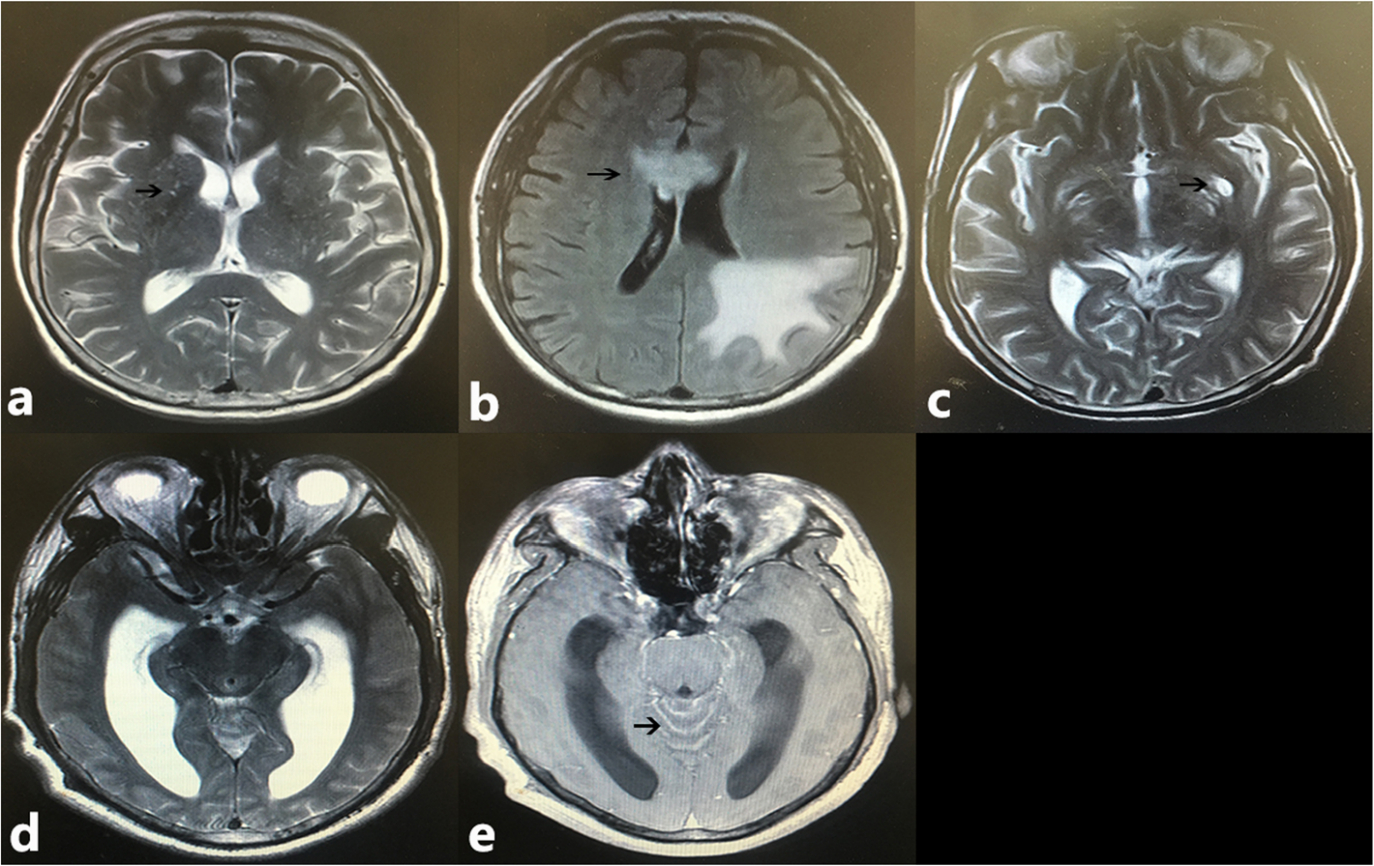
Neuroimaging characters of patients with cryptococcal meningitis. **a** T2-W image shows multiple dilated Virchow-Robin spaces (black arrow) in basal ganglia; **b** Abnormality (black arrow) on FLAIR image within the occipital lobe and corpus callosum; **c** T2-W image shows a gelatinous pseudocyst (black arrow) in basal ganglia; **d** hydrocephalus on T2-W image; **e** Contrast-enhanced image shows meningeal enhancement (black arrow) in cerebellum

MR imaging findings were summarized in Table 2 and Fig. 1. A total of 81.6% (62/76) patients had abnormal image findings. Parenchymal lesions, dilated Virchow-Robin spaces and meningeal enhancement were the three most common findings.

At discharge, we further assessed the outcome for all patients by using GOS, 35 patients (46.1%) obtained a good outcome.

In a univariate analysis comparing the good outcome group with the unfavorable outcome group, non-parametric test confirmed that unfavorable outcome was associated with lower glucose level of CSF(*P* = 0.001), and Pearson’s χ2 analysis confirmed that unfavorable outcome was associated with opening pressure of CSF(>300mmH20, *P* = 0.038), impaired consciousness (*P* = 0.001), hydrocephalus(*P* = 0.045), and Shunt surgery (*P* = 0.045). Tables 3 and 4 summarize the results of further analyses comparing the quantitative and qualitative variables between the good outcome group and the unfavorable outcome group. Multiple logistic regression analysis confirmed that impaired consciousness and glucose concentration of CSF were prognostic factors which predict the outcome in immunocompetent patients with CM (Table 5).

**Table 3:** Results of univariate analysis identifying variables that differed significantly between the good and unfavorable outcome groups

Quantitative variable	Good (*n* = 35)	Unfavorable (*n* = 41)	*P*-value
T-test			
Age at onset (years)	45.63 ± 13.80	52.24 ± 15.20	0.052
CSF opening pressure (mmH2O)	290.46 ± 108.24	328.05 ± 119.80	0.158
Rank sum test			
Interval from onset to antifungal treatment (day)	30.27 ± 25.65	38.69 ± 38.42	0.223
Duration of antifungal treatment (day)	41.61 ± 36.85	32.81 ± 26.43	0.346
Blood WBC count(10^9^/L)	8.96 ± 4.06	10.04 ± 3.85	0.177
CSF WBC count(10^6^/L)	92.71 ± 80.54	77.39 ± 104.71	0.104
CSF Glucose (mmol/L)	2.43 ± 1.22	1.54 ± 1.32	0.001
CSF Chloride (mmol/L)	117.34 ± 4.82	115.81 ± 9.70	0.794
CSF Protein (g/L)	0.96 ± 0.52	1.25 ± 0.93	0.673

Quantitative results are expressed as the mean ± standard deviation. *CSF* Cerebrospinal fluid

**Table 4:** Analysis identifying qualitative variables that differed significantly between the good and unfavorable outcome groups

Qualitative variables	Good (*n* = 35)	Unfavorable (*n* = 41)	*P*-value
Gender (male)	23	27	0.990
Am B administration	33	36	0.565
Shunt surgery	2	9	0.045
Headache	34	37	0.456
Fever	23	25	0.669
Vomiting	19	19	0.490
Impaired consciousness	1	14	0.001
Visual disturbance	5	10	0.270
Seizures	1	8	0.065
Limb weakness	3	4	1.000
Altered mentation	2	5	0.565
Hearing impairment	1	1	0.910
CSF Opening pressure(>300mmH2O)	13	25	0.038
Meningeal irritation positive	17	28	0.081
India ink test and culture positive	15	11	0.142
Dilated VRS	23	27	0.990
Parenchymal lesions	19	22	0.956
Meningeal enhancement	6	10	0.440
Hydrocephalus	2	9	0.045
Gelatinous pseudocyst	3	4	1.000

Pearson’s χ^2^ continuity correction of Fisher’s exact test was used for statistical analysis; *CSF* Cerebrospinal fluid

**Table 5:** Results of backward stepwise multiple logistic regression analysis of variables that differed significantly between the good and unfavorable outcome groups

Variable	Engel classification	
	Regression coefficient	*P*-value
CSF Glucose	−0.575	0.012
Impaired consciousness	2.683	0.015
Constant	0.779	0.130

*CSF* Cerebrospinal fluid

## Discussion

Environmental resource plays an important role in epidemiology of *Cryptococcus neoformans*. Previous studies have discovered that cryptococcosis may developed in people after exposure to birds or bird guano [10, 11]. In the study conducted by XX et al., 20 samples of pigeon droppings (5%,*N* = 400) were positive for *C. neoformans*, and pigeon excreta is determined as a favorable environment for growth of this organism [3]. Besides, Pigeon are popular as pet and are considered as a bird of peace in general population; therefore, they raise and care in close with human living area, which contribute to spread of pathogen *Cryptococcus neoformans*.

CM has often been described as an opportunistic infection in immunocompromised individuals [12, 13]. Yuchong C, et al. collected 7315 cases with CM and discovered that most common underlying diseases were HIV infection, liver disease, systemic lupus erythematosus, and diabetes mellitus, which are correlated with immunodeficiency, only 17% of patients had no underlying diseases [14]. That is why most previous studies have focused on individuals with or without AIDS. In fact, CM has been seen more frequently in immunocompetent individuals in china. The researches from Hongkong and Taiwan show 55–67% of patients are apparently immunocompetent [9, 15, 16], and three studies from mainland china demonstrated a high proportion of immunocompetent patients [17–19]. Consistently, we collected 116 patients identified with CM in the present study, and a high proportion of apparently immunocompetent patients (65.5%) were included. This phenomenon may be explained by the low incidence of AIDS and the lack of full development of organ transplantation in China. Performing Shunt placement reflects the existence of uncontrollable intracranial hypertension that is directly associated with poor clinical manifestation and early death [20–22].

Reviewing the clinical manifestations of the 76 patients in the present study, headache (71/76,93.4%), fever (71/76,93.4%), vomiting (38/76, 50%) were common but not significantly different between the good outcome group and unfavorable groups, almost consistent with previous studies [5, 9].. Several studies focused on non-HIV patients reported the correlation between initial consciousness level and therapeutic outcome [19, 23, 24]. Consistently, in the present study, logistic regression analysis demonstrated that CM patients with impaired consciousness had a significantly higher probability of an unfavorable outcome.

Several previous studies confirm that high CSF opening pressure is prognostic factors independently associated with unfavorable outcome or increased odds of mortality [5, 25], in present study, CSF opening pressure(>300mmH2O) is associated with unfavorable outcome, but our multivariate logistic regression analyses did not identify it as a significant prognostic factor for outcome which may due to small sample size. CSF opening pressure requires further analysis in larger cohorts of immunocompetent patients. Decreased CSF glucose concentration is used as indicators in the process of diagnosing community-acquired meningitis [26]. In previous studies from china, patients with Decreased CSF glucose level tended to have worse outcome [18, 27], and this also occurred in our patient population. Infection with *Cryptococcus neoformans* caused dysfunction of the blood–brain barrier and presentation of cryptococcus in CSF [28], which result in the abnormality of glucose transportation and increased glycolysis, respectively [29]. Those factors lead to decreased CSF glucose level in CM patients.

In a study focused on organ transplant recipients with CM, only sixteen patients (29%/*N* = 55) had abnormal findings [30]. In contrast, more immunocompetent patients (81.6%/*N* = 76) show abnormal MRI findings in our patient population. An autopsy research compared immunocompetent and immunocompromised patients found that lesions were more restricted to the perivascular space [31], and VRS are perivascular spaces at thalamus, basal ganglia, periventricular white matter and the cerebellum [32]. Consistently, Dilated VRS is the most common finding of neuroimaging in our study. But the former research maybe underestimates the presence of parenchymal lesion because of small case size (*N* = 27). In fact, up to 41 patients (53.9%) show parenchymal lesion in our cases, which is in accordance with our previous study focused on neuroimaging findings (61.1%/*N* = 18) [33]. This may be explained by cryptococcal infection that spread into the parenchyma through the VRS during disease progression.

MRI scan is routine and important examination in early diagnosis of CNS infection, we try to find some imaging characters to predict the outcome of CM patients. Hydrocephalus is common abnormal finding of imaging, which has been reported in several studies as an important factor for unsatisfactory outcome in HIV-negative patients with CM [34] and tuberculous meningitis [35], and hydrocephalus is significantly associated with unfavorable outcome in the present study. However, it doesn’t be identified as a predictor Independent prognostic factor, which may be due to too few cases with hydrocephalus. The present study is limited by following factors. First, our study is a retrospective, and single-center design and small sample size may lead to neglect and underestimation of some significant predictors. Second, variant administration of antifungal treatment made it difficult to investigate their influences on outcome. Third, lack of findings about serotypes and varieties of *Cryptococcus neoformans* may result in neglect of more correlation between clinical data and microbiological findings. Forth, clinical outcome was graded by the Glasgow outcome scale at discharge without follow-up. So that prospective clinical researches with a large population, multicenter, and long-term follow up are required in order to yield more reliable results.

## Conclusion

Impaired consciousness and decreased glucose concentration of CSF increase the likelihood of unfavorable outcome in CM patients. Prior research and our findings indicates that absences of Hydrocephalus and CSF Opening pressure(<300mmH2O) are beneficial for achieving a favorable outcome.

## Data Availability

The data that support the findings of this study are available from the corresponding author via E-mail upon reasonable request.

## Abbreviations

CM: Cryptococcal meningitis
CNS: Central nervous system
CSF: Cerebrospinal fluid
GOS: Glasgow outcome scale
HIV: Human Immunodeficiency Virus
VRS: Virchow-Robin spaces
